# Publisher Correction: Real-space observation of ergodicity transitions in artificial spin ice

**DOI:** 10.1038/s41467-023-44006-3

**Published:** 2023-12-04

**Authors:** Michael Saccone, Francesco Caravelli, Kevin Hofhuis, Scott Dhuey, Andreas Scholl, Cristiano Nisoli, Alan Farhan

**Affiliations:** 1 Center for Nonlinear Studies and Theoretical Division, Los Alamos National Laboratory, Los Alamos, NM 87545 USA; 2 Laboratory for Mesoscopic Systems, Department of Materials, ETH Zurich, 8093 Zurich, Switzerland; 3 Laboratory for Multiscale Materials Experiments (LMX), Paul Scherrer Institute, 5232 Villigen PSI, Switzerland; 4 Molecular Foundry, Lawrence Berkeley National Laboratory, One Cyclotron Road, Berkeley, CA 94720 USA; 5 Advanced Light Source, Lawrence Berkeley National Laboratory, One Cyclotron Road, Berkeley, CA 94720 USA; 6 Department of Physics, Baylor University, Waco, TX 76798 USA

**Keywords:** Magnetic properties and materials, Two-dimensional materials, Phase transitions and critical phenomena, Metamaterials

Correction to: *Nature Communications* 10.1038/s41467-023-41235-4, published online 14 September 2023

The original version of the Peer Review File associated with this Article contained 13 pages. The Peer Review File was updated shortly after publication to remove page 13 that had been erroneously added to the original file.

### Supplementary information

Updated Peer Review file

