# Supplementary material for: Real-space observation of ergodicity transitions in artificial spin ice
Source: Nat Commun. 2023 Sep 14;14:5674. doi: 10.1038/s41467-023-41235-4 (PMC10499874; doi:10.1038/s41467-023-41235-4)
Supplement: Supplementary file 1 — Supplementary Information [file 41467_2023_41235_MOESM1_ESM.pdf]

Supplementary Information

Real-space observation of ergodicity transitions in artificial spin ice

Michael Saccone[1*], Francesco Caravelli[1], Kevin Hofhuis[2,3], Scott Dhuey[4], Andreas Scholl[5], Cristiano Nisoli[1], and Alan Farhan[6*]

[1]Center for Nonlinear Studies and Theoretical Division, Los Alamos National Laboratory, Los Alamos, New Mexico 87545, USA.
[2]Laboratory for Mesoscopic Systems, Department of Materials, ETH Zurich, 8093 Zurich, Switzerland.
[3]Laboratory for Multiscale Materials Experiments (LMX), Paul Scherrer Institute, 5232 Villigen PSI, Switzerland.
[4]Molecular Foundry, Lawrence Berkeley National Laboratory, One Cyclotron Road, CA 94720, USA.
[5]Advanced Light Source, Lawrence Berkeley National Laboratory, One Cyclotron Road, CA 94720, USA.
[6]Department of Physics, Baylor University, Waco, Texas, 76798, USA.

Supplementary Figures

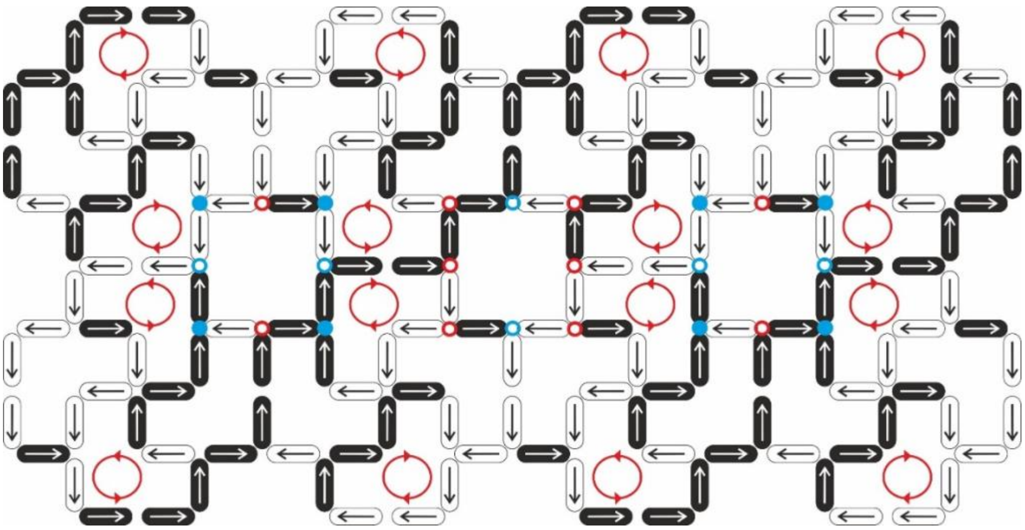

Supplementary Figure 1. Nearest-neighbor ground state. An arrangement of moments that settles each coordination two and four vertex into its local ground state while minimizing the proportion of Type B vertices, visualized by filled blue or red circles, to 25% of coordination three sites. Each coordination four vertex may independently switch orientation along with its adjacent coordination two vertices without changing local dipolar energy, resulting in an extensive bitwise entropy of $s = 1/20$ due to the size of the unit cell containing the coordination four vertex being 20.

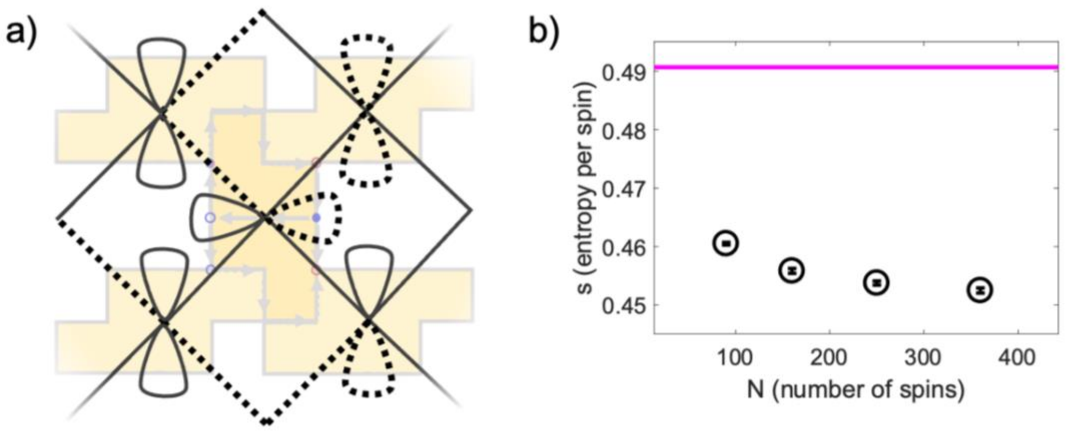

Supplementary Figure 2. Theoretical prediction for the entropy per spin based on the degeneracy of Type B placement. a, A graph comprising a square grid with two loops per node that represents the potential placement of Type B vertices. In the empirically supported model, precisely two edges per node must be selected as Type B vertices. The dotted lines represent one such configuration, corresponding to the spin structure of the greyed out underlying lattice. **b,** Two estimates of the entropy per spin. The pink line is the Pauling upper bound on entropy per bit, $s = \frac{1}{10}\log_2 2\binom{6}{2}$, derived by considering an isolated node. The black circles are brute force estimates of the entropy of graphs corresponding to increasing numbers of spins. The placement of a neighboring Type B vertex may constrain another node's configurations, reducing the total entropy.
