# Supplementary material for: Real-space observation of ergodicity transitions in artificial spin ice
Source: Nat Commun. 2023 Sep 14;14:5674. doi: 10.1038/s41467-023-41235-4 (PMC10499874; doi:10.1038/s41467-023-41235-4)
Supplement: Supplementary file 3 — Description of Additional Supplementary Files [file 41467_2023_41235_MOESM3_ESM.docx]

**Description of Additional Supplementary Files**

**File Name: Supplementary Movie 1
Description:** An XMCD image sequence showing the magnetic moments of Apamea lattice evolving over time at 290 K. Each frame corresponds to a time of 10 seconds between successive images. Black domains point to the left while White domains point to the right.

**File Name: Supplementary Movie 2**

**Description:** An XMCD image sequence showing the magnetic moments of Apamea lattice evolving over time at 300 K. Each frame corresponds to a time of 10 seconds between successive images. Black domains point to the left while White domains point to the right.
